# Change of CGRP Plasma Concentrations in Migraine after Discontinuation of CGRP-(Receptor) Monoclonal Antibodies

**DOI:** 10.3390/pharmaceutics15010293

**Published:** 2023-01-15

**Authors:** Bianca Raffaelli, Maria Terhart, Mira Pauline Fitzek, Kristin Sophie Lange, Jasper Mecklenburg, Lucas Hendrik Overeem, Anke Siebert, Elisabeth Storch, Uwe Reuter

**Affiliations:** 1 Department of Neurology, Charité Universitätsmedizin Berlin, 10117 Berlin, Germany; 2 Clinician Scientist Program, Berlin Institute of Health at Charité (BIH), 10117 Berlin, Germany; 3 Universitätsmedizin Greifswald, 17475 Greifswald, Germany

**Keywords:** monoclonal antibodies, CGRP, migraine, preventive treatment, biomarker

## Abstract

Discontinuation of treatment with monoclonal antibodies (mAb) targeting the Calcitonin Gene-Related Peptide (CGRP) pathway leads to an increase in migraine frequency. We aimed to assess changes in free and total CGRP plasma concentrations after the discontinuation of CGRP(-receptor) mAbs. This prospective analysis included 59 patients with migraine (*n* = 25 erenumab, *n* = 25 galcanezumab, *n* = 9 fremanezumab) who discontinued mAbs after ≥8 months of treatment. Patients were visited at the time of the last mAb injection (V1) and 16 weeks later (V2). For control, 30 migraine patients without preventive drug therapy were included. We measured free CGRP plasma concentrations in the erenumab and fremanezumab group and total CGRP concentrations in the galcanezumab group. Free CGRP plasma concentrations did not change after treatment discontinuation [erenumab: V1 31.2 pg/mL (IQR 25.8–45.6), V2 30.3 pg/mL (IQR 22.9–47.6), *p* = 0.65; fremanezumab V1 29.4 pg/mL (IQR 16.4–61.9), V2 34.4 (19.2–62.0), *p* = 0.86]. Controls had similar CGRP values of 32.6 pg/mL (IQR 21.3–44.6). Total CGRP concentrations in the galcanezumab group were 5439.3 pg/mL (2412.7–6338.1) at V1, and decreased to 1853.2 pg/mL (1136.5–3297.0) at V2 (*p* < 0.001). Cessation of treatment with CGRP(-R) mAbs did not have an impact on the free-circulating CGRP concentrations. Total CGRP decreased significantly after three months of treatment discontinuation.

## 1. Introduction

Calcitonin Gene-Related Peptide (CGRP) is a key neurotransmitter in the development of migraine attacks [1]. During the last decade, CGRP has emerged as a specific therapeutic target for migraine. Monoclonal antibodies (mAbs) targeting the CGRP pathway are approved for migraine prophylaxis in patients with ≥4 monthly migraine days (MMD) [2]. Both the CGRP-receptor (CGRP-R) mAb erenumab and the CGRP mAbs galcanezumab and fremanezumab have demonstrated good efficacy and tolerability in randomized controlled trials and real-world studies [3].

The original expert consensus of the European Headache Federation (EHF) from 2019 on the use of CGRP(-R) mAbs recommended a treatment duration of 6–12 months, followed by a discontinuation attempt [4]. This recommendation was published shortly after the approval of the mAbs, in accordance with the recommendation for unspecific oral preventive medications [4]. Over the years, real-world studies have shown that treatment discontinuation leads to a relevant and continuous increase of migraine frequency [5,6,7]. We previously found a significant worsening of MMD already during the first month of discontinuation [7]. After three months without drug treatment, migraine frequency went back to the level prior to treatment initiation [7].

CGRP concentrations in blood plasma have been proposed as a biomarker for migraine activity [8]. CGRP plasma levels increase during migraine attacks, and return to normal after triptan administration [1]. Some studies detected elevated CGRP plasma levels even in the interictal period, in particular in patients with chronic migraine [9,10]. CGRP in plasma could also serve as a marker for treatment monitoring [11]. Cernuda-Morollón and colleagues described a significant decrease in CGRP plasma levels under successful OnabotulinumtoxinA therapy [11]. CGRP-targeted treatments in particular are thought to have an impact on CGRP plasma concentrations [12]. However, a proof-of-concept and a second study on CGRP in peripheral blood in patients on erenumab remained inconclusive [13,14].

In this study, we aimed to assess the differences of CGRP plasma concentrations during and after cessation of treatment with CGRP(-R) mAbs. We also wanted to determine if patients treated for several months with CGRP(-R) mAbs present different CGRP plasma concentrations than patients without any prophylactic treatment.

## 2. Materials and Methods

### 2.1. Study Design and Participants

This analysis is part of a longitudinal, prospective cohort study conducted at the Headache Center of the Charité—Universitätsmedizin Berlin. The study design has been described in detail elsewhere [7]. In brief, we included patients with a diagnosed episodic migraine (EM) or chronic migraine (CM) [15] who received preventive treatment with a CGRP(-R) mAb and underwent treatment discontinuation after a minimum of eight treatment months. The dose of erenumab was 70 or 140 mg monthly at the discretion of the treating physician. Galcanezumab was administered starting with a 240 mg loading dose, followed by 120 mg monthly, and fremanezumab at a monthly dose of 225 mg. Exclusion criteria were: previous exposure to CGRP-targeted treatment prior to the current treatment cycle; concomitant preventive treatments; other neurological diseases; other headache disorders apart from tension-type headache. Patients were divided into three groups based on the mAb they received (erenumab, galcanezumab or fremanezumab).

For control, we recruited age- and sex-matched patients with EM or CM without any current prophylactic drug treatment.

### 2.2. Study Procedures

For this analysis, patients with mAb treatment had two study visits: at the time of the last mAb injection (V1) and 16 weeks later (V2), when patients had been three months without CGRP(-R) mAb treatment. Controls were measured once at a random time point (V1). All visits of the control group took place during the interictal time, i.e., the patients had been migraine-free for at least 24 h prior to the study visit. In the mAb groups, we adhered to the scheduled date of the last mAb administration and 16 weeks later, respectively. Therefore, ictal visits were permitted.

At each study visit, headache information of the previous four weeks was extracted from standardized headache diaries, as previously described [7]. The headache data of interest included monthly migraine days (MMD), monthly headache days (MHD), and monthly days with use of acute medication (AMD).

We then collected blood samples from the antecubital vein following a standardized protocol [16]. We prepared cooled 10 mL EDTA tubes (BD Vacutainer^®^, Franklin Lakes, NJ, USA) with 500 µL aprotinin (3–7 trypsin inhibitor unit (TIU)/mL) (Sigma Aldrich, Munich, Germany) and drew blood directly in the precooled tubes. The tubes were then immediately centrifuged for 15 min at −6 °C and 2000 rpm. After centrifugation, plasma was extracted and stored at −80 °C.

The analysis of CGRP was performed using a commercial enzyme immunoassay-KIT (EIA) (Bertin Bioreagent, Montigny le Bretonneux, France), which can measure all human CGRP isoforms [17]. This is a two-site immunometric sandwich assay, using an anti-C terminus mAb as the capture antibody and an anti-N terminus mAb as a tracer [17]. The anti-C terminus EIA antibody binds to the same epitope as the commercial CGRP mAb fremanezumab, but to a different epitope than galcanezumab. Therefore, in patients treated with fremanezumab, the used EIA can detect only unbound, free-circulating CGRP. The same applies for patients treated with erenumab, which binds to the CGRP-R. In contrast, in patients on galcanezumab treatment, the EIA detects both free CGRP and the CGRP-galcanezumab complex. The different binding sites are essential for result interpretation. Accordingly, we here provide data on free CGRP for the erenumab, fremanezumab, and control group, and of total CGRP (i.e., free + bound CGRP) for galcanezumab.

### 2.3. Outcomes and Endpoints

The primary endpoint of this analysis was the difference of CGRP plasma concentrations (pg/mL) between V1 and V2 in all (*n* = 3) mAb treatment groups.

Secondary endpoints were the differences of CGRP plasma levels between the mAb groups and the control group. At both study visits, we assessed correlations of CGRP plasma levels with MMD and the time interval (number of days) from the last migraine attack. In addition, we performed correlation analyses between the CGRP plasma levels at V1 and MMD, MHD, and AMD at V2.

### 2.4. Statistical Analysis

Statistical analysis was performed using SPSS 25 (IBM, New York, NY, USA). We summarized all variables of interest using frequencies and percentages for categorical variables, and median (interquartile range, IQR) for numerical variables.

Outcomes were compared using non-parametric procedures due to the non-normal distribution of data. We used the Wilcoxon test for the primary endpoint and the Friedman test for the secondary endpoint. Correlations were tested using the Spearman’s rank correlation coefficient. The significance level was set to *p* = 0.05.

## 3. Results

### 3.1. Demographics and Headache Characteristics

Between January 2020 and February 2021, 80 patients met the criteria for study participation and signed informed consent. Of these, 59 patients agreed to participate in the plasma CGRP measurement study and were included in this analysis. The cohort consisted of n = 25 patients treated for migraine prophylaxis with erenumab, n = 25 with galcanezumab, and n = 9 with fremanezumab. The control group consisted of n = 30 patients without prophylactic drug treatment.

Age, sex distribution, and migraine frequency at V1 were similar in all mAb groups and in the control group (Table 1). In all mAb groups, migraine frequency increased significantly during treatment discontinuation, as reported previously [7] (Table 1).

### 3.2. Determination of Free CGRP Plasma Levels in the Erenumab and Fremanezumab Groups

**Figure 1:**
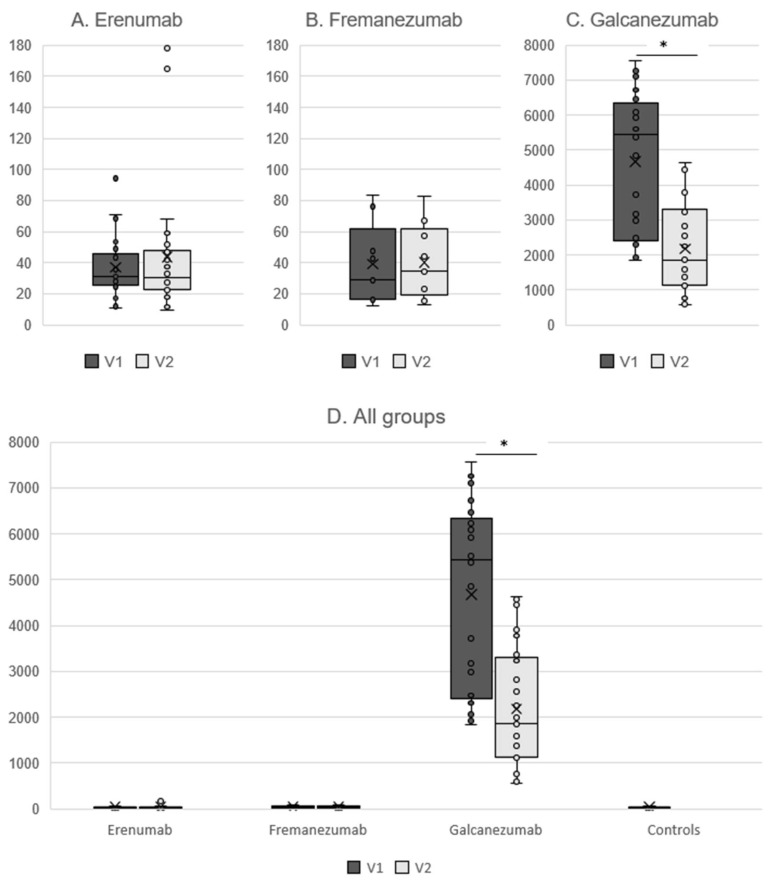
Free CGRP plasma concentrations in pg/mL in patients treated with erenumab (**A**) and fremanezumab (**B**), total CGRP concentrations for galcanezumab (**C**), and comparison between all groups (**D**). V1 = last mAb injection. V2 = 16 weeks after the last mAb injection. * *p* < 0.001.

Free CGRP plasma concentrations in the erenumab group amounted to 31.2 pg/mL (25.8–45.6) at the time of the last mAb injection (V1) and did not change after 16 weeks [V2: 30.3 pg/mL (22.9–47.6), *p* = 0.65] (Figure 1A). Similar concentrations were detected in the fremanezumab group with 29.4 pg/mL (16.4–61.9) at V1 and 34.4 (19.2–62.0) at V2 (*p* = 0.86) (Figure 1B).

We did not find any correlation between the number of MMD and CGRP plasma concentrations in both groups (*p* > 0.54 for all visits). Six patients (17.6%) were in an ictal state during V1 and n = 11 (32.4%) during V2. There was no correlation between the time interval from the last migraine attack and free CGRP plasma concentrations (*p* > 0.27 for all visits). We did not observe any correlation between the CGRP plasma concentrations at V1 and headache parameters (MMD, MHD, AMD) at V2 (*p* > 0.27 for all correlation analyses).

The control group had CGRP plasma concentrations of 32.6 pg/mL (21.3–44.6), which were not different to the erenumab and fremanezumab groups at both time points (*p* > 0.999).

### 3.3. Determination of Total CGRP Plasma Levels in the Galcanezumab Group

Patients in the galcanezumab group had a total CGRP concentration of 5439.3 pg/mL (2412.7–6338.1) at V1, which decreased to 1853.2 pg/mL (1136.5–3297.0) at V2 (*p* < 0.001) (Figure 1C). Total CGRP concentrations did not correlate with MMD (*p* > 0.43 for both visits). V1 and V2 were conducted ictally in n = 5 patients (20.0%) and n = 7 patients (28.0%), respectively. The time interval from the last migraine attack had no significant correlation with the total CGRP levels (*p* > 0.16 for both visits). There was no correlation between the total CGRP concentration at V1 and MMD/MHD/AMD at V2 (*p* > 0.25 for all correlation analyses).

Due to the assay properties, total CGRP concentrations in the galcanezumab group were much higher than the free CGRP concentrations in all the other groups (*p* < 0.001) (Figure 1D).

## 4. Discussion

This prospective cohort study shows that concentrations of free-circulating plasma CGRP in migraine patients do not change after the cessation of an eight-month prophylaxis with erenumab and fremanezumab. The amount of free-circulating CGRP in plasma in patients on erenumab and fremanezumab treatment and after discontinuation was similar to migraine patients without any preventive treatment. Total CGRP concentrations were elevated under galcanezumab treatment and decreased after treatment interruption.

Kielbasa and Helton suggested in the pharmacokinetic/pharmacodynamics model of galcanezumab that free CGRP concentrations decrease rapidly within the first day after mAb administration and then slowly return to pre-treatment levels [12]. The binding of CGRP to a mAb prevents CGRP from rapid degradation. CGRP-mAb complexes have similar pharmacokinetic features to the mAb alone, with an elimination half-time of approximately four weeks [12]. This is in line with our findings on total CGRP in patients treated with galcanezumab: total CGRP levels at V1 were about 200× higher than the free CGRP concentrations in the other groups and slowly diminished after treatment cessation. The clinical significance of the circulating CGRP-mAb complexes after treatment discontinuation remains to be determined. They do not seem to have an impact on migraine frequency, since the number of MMD at V2 was similar to the levels prior to treatment beginning [7].

A simulation model under steady state administration of 120 mg galcanezumab after a 240 mg loading dose revealed a 61% decrease of free CGRP from baseline [12]. We do not have data on free CGRP plasma levels under galcanezumab treatment, but our data on fremanezumab does not match this theoretical simulation. Free CGRP plasma concentrations after at least eight months of fremanezumab treatment were similar to those of patients without any preventive treatment. To our knowledge, data on CGRP plasma levels in patients with migraine treated with a CGRP mAb have not yet been published. For erenumab, one small study with seven patients detected numerically higher CGRP plasma levels after six months of treatment but without statistical significance [13]. The authors proposed an upregulation of CGRP following the blockade of the CGRP-R [18]. In our larger cohort of patients treated with erenumab, free CGRP plasma levels did not differ from those of prophylaxis-naïve patients and remained stable after treatment discontinuation. In line with our results, a recent study on serum CGRP concentrations did not show any difference between before and 2–4 weeks after starting erenumab treatment [14]. Similarly, a study on salivary CGRP levels detected no significant change during 12 weeks of erenumab therapy [19]. In contrast to migraine frequency, free CGRP plasma concentrations do not appear to change under CGRP(-R) treatment or after discontinuation. Accordingly, they do not seem suitable as a therapeutic biomarker in this case.

The lack of changes in the free CGRP plasma concentrations after several months of treatment with CGRP(-R) mAbs could provide relevant safety information about these novel preventive therapies. CGRP is the most potent vasodilator in the human body and is ubiquitously expressed throughout the nervous system [20]. Among several other functions, CGRP has a protective role in the cardiovascular system [21,22]. It serves as a defense mechanism during pathological conditions such as cerebral or myocardial infarction [23]. The long-term blockade of the CGRP pathway via mAbs has raised concerns among experts about the cardiovascular safety of these drugs [21]. Our analysis indicates that free CGRP plasma concentrations remain in a comparable range to those of migraine patients without treatment. We speculate that the mAbs might prevent CGRP spikes that lead to acute migraine attacks without affecting the basal CGRP concentrations to a relevant degree. This speculative hypothesis should be confirmed in further studies, e.g., comparing ictal CGRP concentrations during mAb treatment compared to the time prior to treatment begin. In line with this hypothesis, clinical trials showed a comparable rate of cardiovascular adverse events between CGRP(-R) mAbs and the placebo [24,25]. However, a recent real-world study detected increased blood pressure values in patients treated with erenumab and fremanezumab [26]. Although the increase in blood pressure was not clinically relevant in most patients, the risk of hypertension while blocking the CGRP pathway should be carefully considered. Future studies should assess if blood pressure values change after treatment discontinuation and if there is any association between blood pressure and CGRP plasma levels.

This is the first longitudinal analysis examining total and free CGRP plasma values in patients treated with CGRP(-R) antibodies and after their discontinuation. For CGRP analysis, we followed an established protocol [16]. Degradation of CGRP was minimized by pre-analytical vial preparation with a protease inhibitor as well as immediate sample processing and cooling. The feasibility of CGRP measurement in peripheral blood as a marker of migraine activity has been a matter of debate [27,28]. In blood plasma, CGRP is subject to dilution, and alternative biomaterials such as saliva or tear fluid are more likely to reflect the trigeminovascular CGRP release [27]. However, the aim of this study was to assess systemic CGRP changes in patients treated with CGRP(-R) mAbs. Therefore, we consider peripheral blood an appropriate medium for this purpose. The main limitation of this study is the lack of baseline CGRP values prior to treatment beginning. We used age- and sex-matched control migraine patients as a surrogate. An intra-individual comparison over all time points would increase data quality and should be aimed for in future studies. Moreover, our findings apply for a mAb treatment duration of 9 months in median and may not be generalizable to significantly longer or shorter treatment periods. Finally, not all patients could be measured in the interictal period. This is not likely to pose a relevant bias in data, since there was no correlation between CGRP levels and the time since the last migraine attack.

In conclusion, patients treated for at least eight months with CGRP(-R) mAbs had similar concentrations of free-circulating plasma CGRP to patients without any preventive treatment. Migraine worsening after three months of treatment discontinuation was not associated with changes in free CGRP. The concentrations of total CGRP were very high under CGRP mAb treatment and decreased slowly after treatment discontinuation.

## Figures and Tables

**Table 1:** Demographic and headache characteristics of study participants at the time of the last mAb injection (V1) and after 16 weeks (V2).

	Erenumab	Galcanezumab	Fremanezumab	Control	*p* ^a^
*n*	25	25	9	30	
Age (years)	52.0 (42.5–57.5)	51.0 (39.5–57.5)	54.0 (50.5–58.5)	52.0 (45.3–56.3)	0.73
Female sex	23 (92%)	24 (96%)	9 (100%)	29 (97%)	0.74
Treatment duration (months)	9.0 (9.0–10.5)	9.0 (9.0–10.0)	9.0 (9.0–10.8)		
MMD at V1	8.0 (4.0–11.0)	5.0 (3.0–12.0)	4.0 (4.0–7.5)	7.3 (5.0–11.0)	0.20
MMD at V2	14.0 (9.5–19.5)	11.0 (7.0–16.0)	6.0 (4.5–17.0)		0.15
p V1 vs. V2	<0.001 *	<0.001 *	0.012 *		
MHD at V1	8.0 (4.0–12.5)	6.0 (3.0–13.0)	4.0 (4.0–8.0)	8.0 (5.8–12.0)	0.39
MHD at V2	14.0 (10.0–19.5)	11.0 (7.0–16.0)	6.0 (4.5–17.0)		0.12
p V1 vs. V2	<0.001 *	<0.001 *	0.068		
AMD at V1	7.0 (2.5–8.0)	5.0 (3.0–8.0)	4.0 (3.5–5.0)	7.0 (4.0–9.0)	0.20
AMD at V2	8.5 (6.0–14.0)	7.0 (4.5–14.0)	6.0 (4.0–8.5)		0.50
p V1 vs. V2	<0.001 *	<0.001 *	0.024 *		

Values are n (%) or median (IQR). Abbreviations: MMD = monthly migraine days; MHD = monthly headache days; AMD = monthly days with acute medication use. ^a^ *p* values for the comparisons between groups. * statistically significant.

## Data Availability

The datasets used and/or analyzed during the current study are available from the corresponding author on reasonable request.
